# The perception of the French /s/-/ʃ/ contrast in early Creole-French bilinguals

**DOI:** 10.3389/fpsyg.2014.01200

**Published:** 2014-10-22

**Authors:** Sophie Dufour, Sibylle Kriegel, Muhsina Alleesaib, Noël Nguyen

**Affiliations:** ^1^Aix-Marseille Université, CNRS, LPL, UMR 730913100 Aix-en-Provence, France; ^2^Brain and Language Research Institute, Aix-Marseille UniversitéAix-en-Provence, France; ^3^Structures Formelles du Langage, CNRS, Université Paris 8Paris, France

**Keywords:** speech perception, bilingualism, Mauritian creole, social characteristics, exemplar models

## Abstract

One particularity of the Mauritian Creole language is that there is no contrastive distinction between the consonants /s/ and /ʃ/, which are both pronounced /s/ in Creole. In this study, we examined the identification performance of the /s/-/ʃ/ contrast by Mauritian Creole-French bilinguals who have been exposed to French before 7 years of age, and who have been raised in a highly Creole-French bilingual society. The results showed that most of our bilingual participants identify the /s/ and /ʃ/ consonants like native French speakers. It also appeared that the way in which the two consonants are categorized can be manipulated by introducing subtle changes in the information these participants were given about the identity of the speaker that produced the stimuli. Our results are in accordance with recent studies showing native-like performance in bilinguals on a categorization task and, importantly, extend these findings to speakers of a Creole language. In addition, these results show that speech sound categorization can be influenced by information about the speaker's social identity and thus argue for models that postulate rich speech sound representations.

## Introduction

Several decades of research show that the way speech sounds are perceived and categorized is influenced by a large number of factors that are both speaker- and listener-dependent. As is well known, the listeners' native language acts as a filter through which they perceive speech sounds, and this often leads to poor performance in the discrimination of non-native consonant and vowel contrasts even in fluent bilingual speakers (e.g., Goto, 1971; Werker and Tees, 1984; Pallier et al., 2001). Also, a growing body of research shows that listeners' perception is influenced by their beliefs about the individual and social identity (e.g., gender, age, social class, regional variety) of the speaker (see, among others, Niedzielski, 1999; Strand, 1999; Hay et al., 2006; Hay and Drager, 2010; Drager, 2011). In the light of prior research on bilingual speech perception, on the one hand, and the effects of the perceived speaker's individual and social identity on how the speaker's speech is processed by listeners, on the other hand, the present study had two goals. First, we examined the identification of the /s/-/ʃ/ French contrast by Mauritian Creole-French bilinguals who have started learning French from an early age, within the first 7 years of their life, and who have been raised in a highly Creole-French bilingual society. Whereas standard French contains a contrastive distinction between /s/ and /ʃ/, this distinction does not exist in Mauritian Creole which only has /s/ for both /s/ and /ʃ/ in standard French word forms. For example speakers of standard French pronounce the words *sac* “bag” and *chaque* “every” as /sak/ and /ʃak/, respectively, whereas speakers of Mauritian Creole use the word form /sak/ for both words. Second, we tested whether the way in which Mauritian Creole listeners categorize these two consonants can be modified by introducing small changes in the information these speakers were given about the identity of the speaker that produced the stimuli.

It is a long-held view that we hear non-native speech sounds in reference to our native phonemic inventory. As a result, most individuals have difficulties in the discrimination of some non-native phonemic contrasts. The most studied case is the poor performance of Japanese speakers in discriminating the English /l/-/r/ contrast. Japanese speakers have been repeatedly shown to have difficulties in discriminating these phonemes without specific training (e.g., Strange and Dittmann, 1984; Logan et al., 1991) and tend to identify both /l/ and /r/ as their own /r/ phoneme. Difficulties have also been reported in English-speaking adults with Hindi (Werker et al., 1981), Salish (Werker and Tees, 1984), and Czech (Trehub, 1976) phonemic contrasts.

Adaptation of the speech processing system to the phonological regularities of the native language has been found to arise very early in childhood, during the first year after birth. By the age of 10–12 months, infants are already less sensitive to non-native phonemic contrasts than to native contrasts (see Best, 1994; Werker, 1994; Pallier et al., 1997b, for reviews). However, decline in sensitivity does not extend to all non-native contrasts. For example, Best et al. (1988) reported that both 14-month-old and adult English-speaking listeners were able to discriminate Zulu click contrasts. According to Best et al. (1988), adult listeners retain the ability to discriminate a non-native contrast when this contrast is unlikely to be perceptually assimilated to one single native phonemic category. If, however, the non-native sounds are both similar to one phoneme of the native language, then discrimination is difficult.

Somewhat surprisingly, it has been shown that even fluent bilingual speakers may experience difficulty in perceiving vowel and consonant contrasts in their second language (L2). In several studies, Pallier et al. (1997a, 2001) investigated whether bilinguals can master the specific phonetic categories that correspond to the two languages equally well. For example, Catalan has two vowels that differ in height, mid-high /e/ and mid-low /ε/, whereas Spanish only has /e/. Using the repetition priming paradigm, Pallier et al. (2001) showed that Spanish speakers who have learned Catalan before 6 years of age processed Catalan words such as /perə/ and /pεrə/ as homophones. Also, using a classification task, along the /e/-/ε/ continuum, Pallier et al. (1997a) showed that the performance of Spanish/Catalan bilinguals to map the stimuli on the two words /perə/ and /pεrə/ was not above the chance level. A subsequent study (Sebastián-Gallés et al., 2005) has shown that even simultaneous Spanish-Catalan bilinguals, who had been exposed to both Spanish and Catalan from birth, retain some difficulties in perceiving the /e/-/ε/ contrast, at least in tasks that tap into lexical representations. According to Flege and Mackay (2004), an early age of L2 acquisition does not guarantee a native-like perception of L2 phonemes. Only bilinguals who both learn their L2 at an early age and mostly use their L2 may reach the performance level of native speakers in L2 perception. In a more recent study, Sundara and Polka (2008) used an AXB task to compare the discrimination of Canadian French and Canadian English coronal stops by simultaneous bilinguals, bilinguals who were exposed to French at 5–6 years of age, and monolingual listeners. The authors showed that simultaneous bilinguals did not differ from monolingual English listeners in discriminating coronal stops, whereas bilinguals who started learning French at 5–6 years of age did. Such an observation suggests that the timing of acquisition is an important factor in the development of phonemic representations corresponding to the two languages. The same conclusion was drawn from studies examining bilingual speech production (e.g., Guion, 2003).

In addition to the listener's native phonemic inventory, the individual and social characteristics that the listeners attribute to the speaker exert a strong influence on the way speech sounds are processed (e.g., Niedzielski, 1999; Strand, 1999; Hay et al., 2006). In a study where video clips of male or female speakers were simultaneously presented with gender-ambiguous tokens from an auditory /s/-/ʃ/ continuum, Strand (1999) showed that participants modified their perception of the fricative depending on the sex of the speaker that they saw, shifting the /s/-/ʃ/ boundary to lower frequencies for male faces and to higher frequencies for female faces. In the same line, Niedzielski (1999) showed that the dialect area attributed to the speaker also affects speech perception. In both Detroit and Canada, speakers produce variants of the diphthong /aw/ with a raised nucleus. Generally, speakers from Detroit associate the raised variants with Canadians and are not aware that they also produce them. Niedzielski (1999) used recordings of a Detroit speaker's raised /aw/ variant, and found that listeners in Detroit “hear” more Canadian Raising when they think that the talker was from Canada rather than Detroit. In a more recent study, Hay et al. (2006) showed that both the perceived age and perceived social class of the speaker affect perception of vowels that are merging. In New Zealand English, the diphthongs /iə/ and /eə/ as found in words such as *near* and *square* are undergoing a merger such that older speakers and individuals of higher social classes maintain a distinction between /iə/ and /eə/, whereas /iə/ and /eə/ have almost merged toward the /iə/ vowel in younger speakers and individuals of lower social classes. Hay et al. (2006) found that New Zealand participants were able to better identify the two vowels if they were shown a photograph of an older individual or someone who was dressed as though he/she was from a high social class. Hence, it appears that implicit knowledge of how phonological variation is socially distributed influences the perception and discrimination of speech sounds.

The observation that the speaker's idiosyncratic and social characteristics affect speech perception is in accordance with exemplar models of speech perception (e.g., Pierrehumbert, 2001; Johnson, 2006). Within these models, each encountered utterance associated with a particular word is stored in the listener's mind as a separate exemplar. Each exemplar includes fine-grained phonetic details, but also information relative to the gender, age, and regional variety, among other characteristics, of the speaker who produced it. The storage of phonetic exemplars is supported by the growing body of research that shows frequency effects among exemplar representations (e.g., Connine, 2004; Connine et al., 2008). The finding that speech perception is influenced by the perceived characteristics of the speaker (e.g., Hay et al., 2006) argues for social indexing of exemplars. Hence, exemplar models appear the best-suited approach to account for the wide range of observations showing listeners' sensitivity to both fine-grained phonetic details and speakers' social characteristics.

In the present study, we examined the perception of the French /s/-/ʃ/ contrast by early Creole-French bilinguals who have been exposed to French before 7 years of age and who have lived in Mauritius since birth. Mauritian Creole is a French-based/lexified Creole language, and is the product of a complex language contact situation mainly between dialectal spoken French, Malagassy and Eastern Bantu languages that started with French colonization in 1721 (for a brief survey, see Baker and Kriegel, 2013). One of the main phonological differences between French and Mauritian Creole and more largely all French Creoles spoken in the Indian Ocean concerns the loss of the opposition between /s/ and /ʃ/, and between /z/ and /ӡ/, reduced to /s/ and /z/ in the Indian Ocean French Creoles (Chaudenson, 1974). For example, Mauritian Creole speakers usually do not distinguish between the consonants /s/ and /ʃ/, which are both pronounced /s/. According to previous work, the absence of these contrasts in today's Mauritian Creole may be due to the fact that they are not always clearly produced in the French dialects of Normandy, and more specifically that spoken by most of the French colonizers in the 18th century. Also, the two contrasts are reduced to /s/ and /z/ in Malagasy, the main substrate language of Mauritian Creole (e.g., Chaudenson, 2003, 2007). Interestingly, recent corpus-based studies (Kriegel et al., in press) indicate that while monolingual speakers of Creole tend to systematically pronounce /s/ in contexts where the corresponding French item would have been /ʃ/, speakers of urban Creole varieties in contact with French, like a bank employee with a French patronym, alternate between /s/ and /ʃ/ in the same phonetic environments. Given such an observation, we also examined whether the categorization of the French /s/ and /ʃ/ consonants by Creole-French bilinguals can be modified when these bilinguals think that they are faced with the speech of someone who frequently speaks French.

The categorization task in which participants had to classify a word-initial phoneme as one of the two standard French consonants /s/ and /ʃ/ was used. The stimuli formed 11 equally-spaced points along an acoustic continuum ranging from /s/ to /ʃ/, and were followed by the sequence /ak/ with which they formed the word *sac* /sak/ at the /s/ endpoint, and the word *chaque* /ʃak/ at the /ʃ/ endpoint. We asked two main questions. First, do early Creole-French bilinguals discriminate the /s/-/ʃ/ contrast as efficiently as French listeners do? In such a case, the percentage of *chaque* /ʃak/ response should show a typical, sigmoid-like increase from the /s/ to the /ʃ/ endpoint of the continuum. If, however, discriminating the /s/-/ʃ/ contrast proves difficult for the bilinguals, we may expect to find a pattern of responses close to the chance level (50% of *chaque* /ʃak/ responses and 50% of *sac* /sak/ responses throughout the continuum). Second, we asked whether the simple fact of giving a very short piece of information about the speaker's identity suffices to modify the categorization of the /s/ and /ʃ/ consonants in Creole-French speakers.

Three groups of participants were tested on the same auditory stimuli. The first group was made up of French speakers and the two other groups were formed by Creole-French bilinguals. The French speakers group and one of the two groups of Creole-French bilinguals (referred to as the no-information group hereafter) performed the identification task with no information about the identity of the speaker that produced the stimuli. The comparison between the French speaker group and the no-information Creole-French bilingual group allowed us to examine whether Creole-French bilinguals perceive the /s/-/ʃ/ contrast like native French speakers do. The other group of Creole-French bilinguals (referred to as the with-information group hereafter) also performed the identification task, but they were told that the stimuli had been recorded by “Mr. Mathieu, an employee of the MCB Bank in Port-Louis.” The comparison between the first and second group of Creole-French bilinguals allowed us to examine whether a very short piece of information about the identity of the speaker can modify the representation that the listeners form about that speaker, which in turn could bias the participants' responses toward the /ʃ/ consonant. Although we can only speculate on the representation that participants created about the speaker, we expected that the French name “Mathieu” in combination with a socially valued job and an urban environment, Port-Louis being the capital of Mauritius, led to a representation of someone that frequently speaks French and thus pronounces both the /s/ and /ʃ/ consonants. As a result, more /ʃak/ responses are expected in the with- than in the no-information group.

## Methods

### Participants

Fifteen French speakers from Aix-Marseille University and 30 Creole-French bilinguals participated in the experiment. Our Mauritius participants were born in Mauritius, and they all had lived in Mauritius since birth. At the time of testing, they were all undergraduate students at the University of Mauritius, where most of the lectures are given in French. All had received a bilingual education, and they were highly proficient speakers of both Mauritian Creole, their native/first language, and French, with exposure to French starting in early childhood, before 7 years of age. The Creole-French bilinguals were split into two groups of 15 participants. The two groups were as closely matched as possible on age of exposure to French. One participant in each group had been exposed to French since birth. The other participants had been exposed to French between 2 and 6 years of age in kindergarten or primary school. The mean age of exposure to French was 3 (range: 0–6) and 3.4 (range: 0–6) for the first and second group, respectively [*t*_(28)_ = 0.67, *p* > 0.20]. The three groups were matched on age [*F*_(2, 42)_ = 1.53, *p* > 0.20]. The mean age was 22.1 years for the first group and 21.5 years for the second group^1^. The mean age for the French speakers group of Creole-French bilinguals was 22.4 years. All participants reported no hearing or speech disorders.

### Materials and procedure

The material was made up of 11 acoustic stimuli equally spaced on a continuum between sac [sak] and chaque [ʃak]. These stimuli were derived from one natural token of *sac* and one natural token of *chaque* recorded by a native French trained phonetician (the last author) in a soundproof room using high-quality recording equipment at a sampling frequency of 22050 Hz. The fricative noise for [s] and that for [ʃ] were extracted from these two tokens and used for constructing an 11-point [s]-[ʃ] sequence by means of a procedure developed by Stevenson (1979) and Repp (1981; see also McQueen, 1991). The 11 fricative noises all had the same duration (183 ms). Each stimulus was then combined with the sequence [ak] recorded by the same speaker. Peak amplitude was normalized so as to be identical for all of the stimuli.

Stimuli were presented binaurally over headphones at a comfortable listening level. Each stimulus (stimulus 1 to stimulus 11) was presented 9 times, in a randomized order, making thus a total of 99 stimuli by participant. After each stimulus, participants had to decide if it was the word *sac* or the word *chaque*. The two words were presented in writing in the instructions. For the with-information group of Creole-French bilinguals, it was also specified in the instructions that the stimuli had been recorded by “Mr Mathieu an employee of the MCB bank in Port-Louis.” For the no-information group of Creole-French bilinguals as well as for the French speakers group, the instructions contained no indication about the identity of the speaker that had produced the stimuli. All participants gave their responses using a button box placed in front of them. The leftmost button was labeled *sac* and the rightmost button was labeled *chaque*.

## Results

### Comparison between French speakers and no-information Creole-French bilinguals

**Figure 1:**
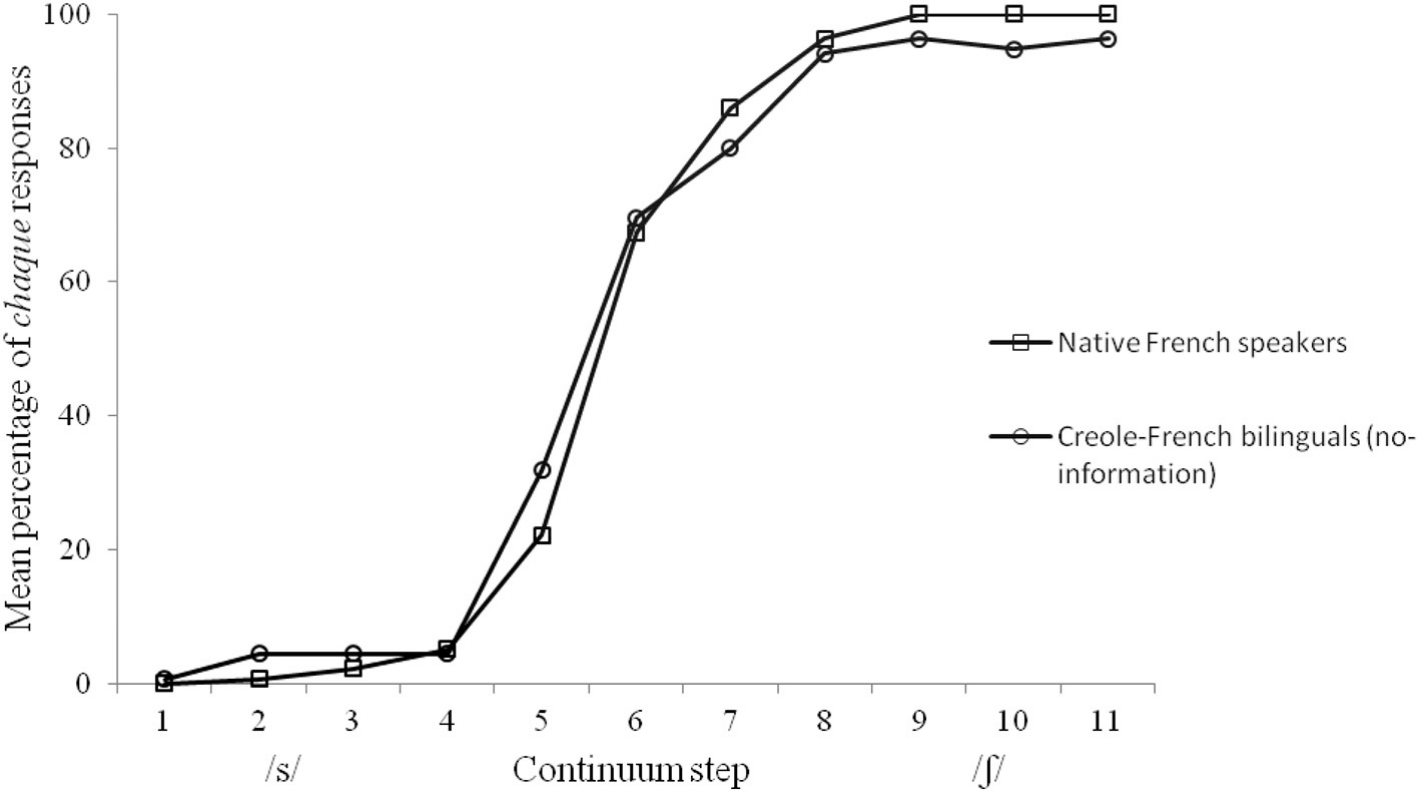
**Mean percentage of *chaque* responses for each stimulus of the /s/-/ʃ/ continuum in the Native French speakers group and in the Creole-French bilinguals group (no-information)**.

The mean percentage of *chaque* responses for the French and the no-information Creole-French bilingual speakers are displayed in Figure 1. An analysis of variance (ANOVA) with step as within-participant factor and group as between-participant factor was performed. Only a main effect of step was observed [*F*_(10, 280)_ = 284.65, *p* < 0.0001] with more *chaque* responses at the /ʃ/ endpoint of the continuum. Neither the main effect of group [*F*_(1, 28)_ = 0.01, *p* > 0.20], nor the interaction between group and step [*F*_(10, 280)_ = 0.76, *p* > 0.20] was significant^2^.

**Figure 2:**
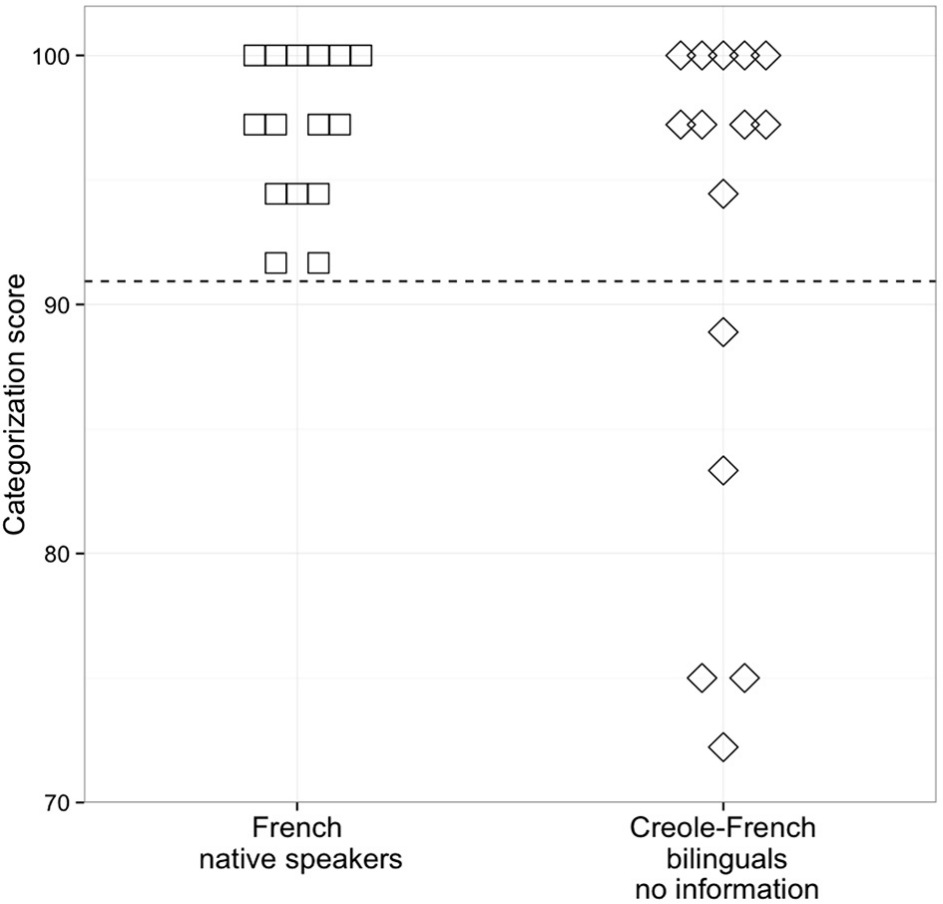
**Individual categorization scores (in %) for Native French speakers and Creole-French bilinguals (no-information)**. The dotted line indicates the native French speakers' cut-off point (2 standard deviations below the mean categorization score).

These first analyses revealed that the performance of our Creole-French bilinguals on a categorization task was similar to that of native French speakers. Because individual differences in mastering L2 phonology have been reported in early and highly proficient bilinguals (e.g., Sebastián-Gallés and Baus, 2005; Sebastián-Gallés and Díaz, 2012), a second analysis was conducted to examine whether all of our bilinguals performed like the native French speakers. We first assessed individual differences by calculating a categorization score for each participant. This score was obtained by averaging the percentage of *chaque* responses for stimuli 1–4, on the one hand, and for stimuli 8–11, on the other hand, thus pooling the endpoints of continuum for which the stimuli were non-ambiguous (see Sebastián-Gallés and Baus, 2005; Díaz et al., 2012). The percentage of *chaque* responses for stimuli 1–4 was then subtracted from the percentage of *chaque* responses for stimuli 8–11. Figure 2 shows that our French speakers have a categorization score comprised between 92 and 100%, and that our Creole-French bilingual speakers have a score comprised between 72 and 100%. In order to evaluate to what extent our bilinguals performed like French native speakers, we then calculated the native performance range by subtracting two standard deviations from the mean categorization score of French native speakers. The resulting cut-off point was 90.94 (mean: 97.04; *sd* = 3.05). As shown in Figure 2, five Creole-French bilinguals (33%) have a score below this cut-off, and thus were outside the native performance range. The categorization score obtained by these five bilinguals was nonetheless comprised between 72 and 89% (i.e., percentage of *chaque* responses comprised between 3 and 14% for stimuli 1–4, and between 78 and 94% for stimuli 8–11). Hence, even if their categorization score was below that of the native French speakers, it was nonetheless high, thus showing that these 5 bilinguals discriminated rather well the /s/ and /ʃ/ consonants.

### Comparison between the no-information and with-information Creole-French bilinguals^3^

**Figure 3:**
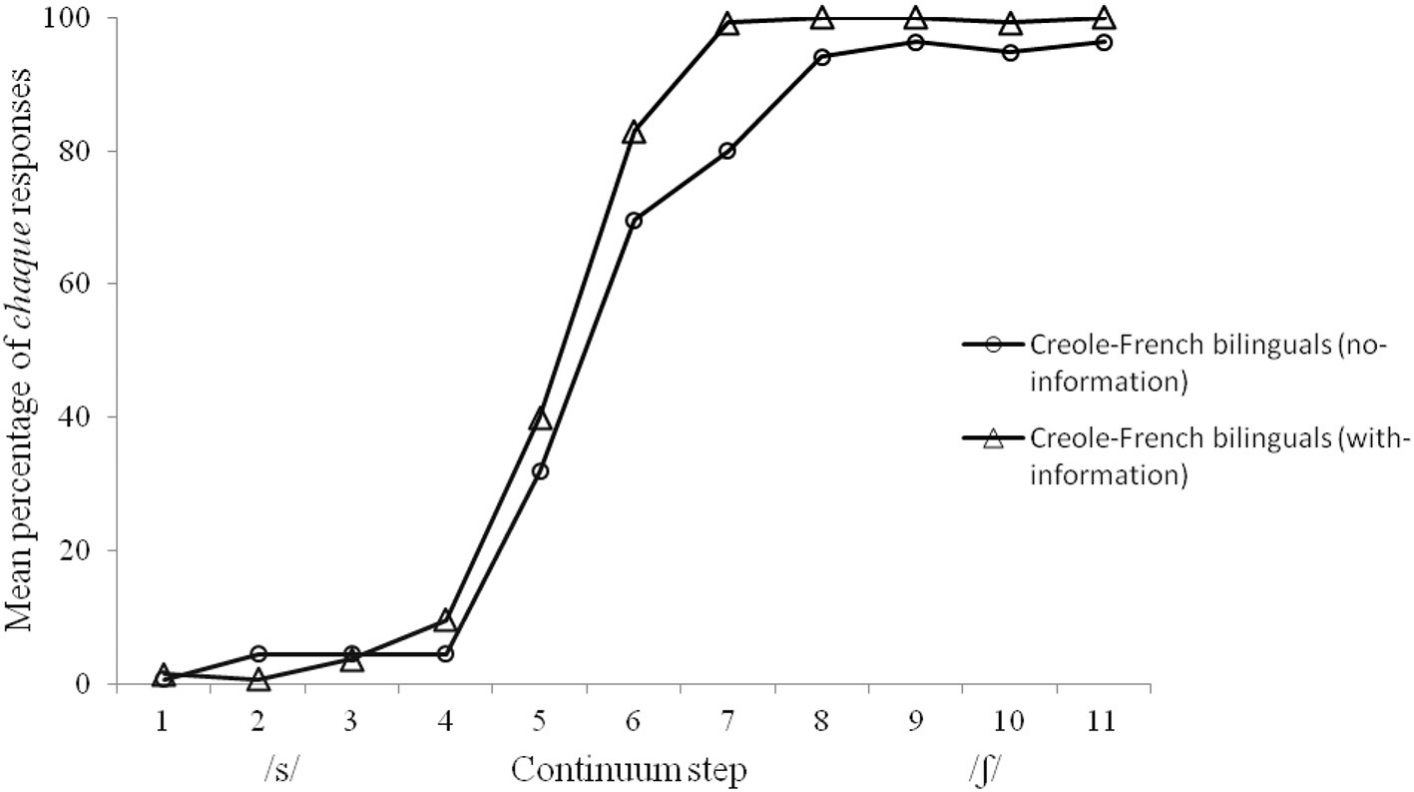
**Mean percentage of *chaque* responses for each stimulus of the /s/-/ʃ/ continuum in the no-information and with-information Creole-French bilinguals**.

**Figure 4:**
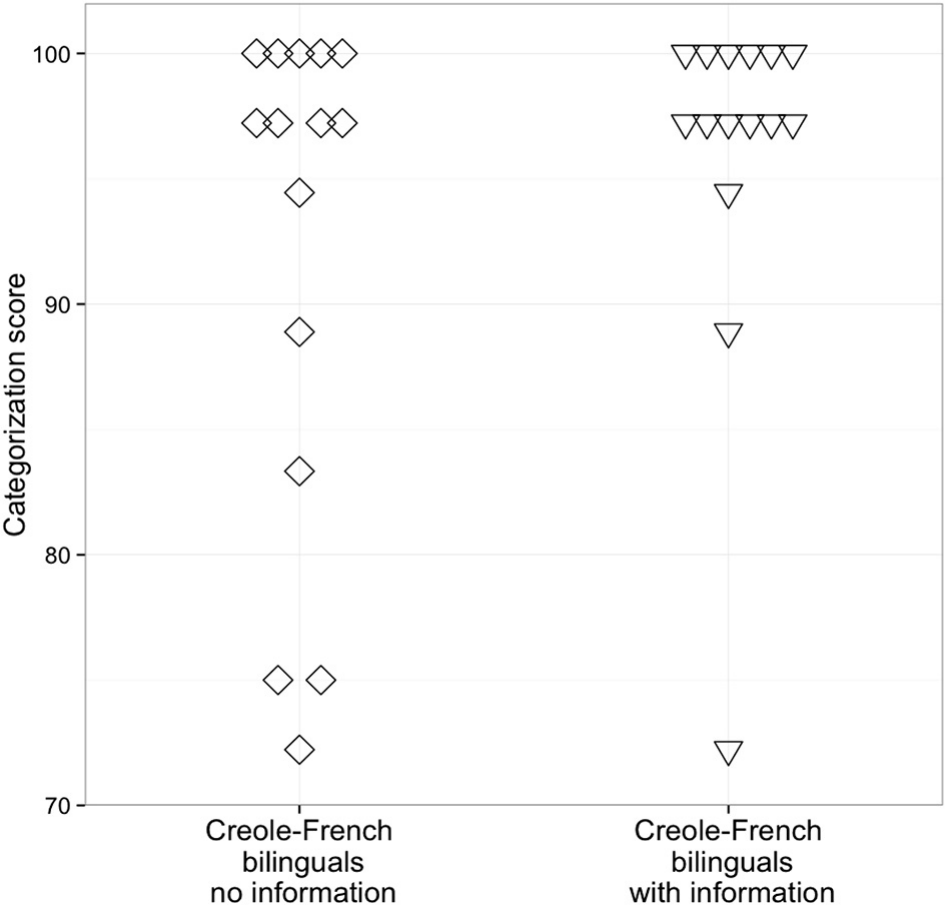
**Individual categorization scores (in %) for the two groups of Creole-French bilinguals (no- and with- information)**.

The mean percentage of *chaque* responses for the two groups of Creole-French bilingual speakers (no- and with- information) are displayed in Figure 3, and the individual categorization scores at the endpoints of the continuum are shown in Figure 4. An ANOVA with step as within-participant factor and group as between-participant factor was performed. As shown in Figure 3, a trend effect of group was observed with a higher number of *chaque* responses in the with-information group than in the no-information group [*F*_(1, 28)_ = 4.07, *p* = 0.05]. The main effect of step was significant [*F*_(10, 280)_ = 300.24, *p* < 0.0001] with more *chaque* responses at the /ʃ/ endpoint of the continuum. To examine in more detail the differences in the percentage of *chaque* responses between the two groups on each step of the continuum, we conducted a series of *t*-tests, with a Bonferroni-corrected alpha level of 0.004. A significant difference between the no-information and the with-information groups on the middle of the continuum [step 7; *t*_(28)_ = 3.26, *p* < 0.004] was observed^2^.

## General discussion

In this study, we tested how well early Creole-French bilinguals perceive and discriminate the French /s/-/ʃ/ contrast, by means of a categorization task. Globally, our results showed that Creole-French bilinguals are readily able to map the stimuli onto the two words *sac* and *chaque*. In particular, our Creole-French bilingual speakers had no difficulty in identifying the four extreme stimuli (i.e., the two contiguous stimuli at either of the two endpoints) as being clearly associated with *sac* on the one hand and *chaque* on the other hand. Additional analyses testing individual differences revealed that only five bilingual participants among the 15 gave fewer *chaque* responses than native French speakers. As noted in the Results Section, their categorization score was however high, thus indicating that these participants also clearly perceived the /s/-/ʃ/ contrast. In contrast, the other 10 Creole-French bilingual participants fell within the performance range of native French speakers, and thus showed native-like performance.

The overall pattern of our results suggests that early Creole-French bilingual speakers can distinguish the French /s/-/ʃ/ contrast as efficiently as native French speakers do. At first sight, our study appears in contradiction with past studies showing systematic difficulties in perceiving L2 phonemic contrasts even for bilinguals that have been exposed to their L2 before 6 years of age and are highly experienced in their L2 (e.g., Pallier et al., 1997a, 2001; Sebastián-Gallés et al., 2005). However, a study conducted by Sebastián-Gallés and Soto-Faraco (1999) with Spanish-Catalan bilinguals shows that these difficulties do not extend to all phonemic contrasts absent in L1. Spanish-Catalan bilinguals performed worse than Catalan-Spanish bilinguals on the /e/-/ε/, /o/-/ɔ/, and /ʃ/-/ӡ/ contrasts, but the performance was similar in the two groups for the /s/-/z/ contrast. All these contrasts are present in Catalan, but not in Spanish. According to the authors, one possible explanation for the good performance on the /s/-/z/ contrast by Spanish-Catalan bilinguals is that /s/ in Spanish is subject to variation and is always realized as /z/ before a voiced consonant. An analysis of the consonants of Mauritian Creole (Baker and Kriegel, 2013) in fact shows that the /ʃ/ consonant is sometimes realized by Mauritian Creole speakers. There are indeed some non-French words, mostly English loan words, in Mauritian Creole vocabulary containing the affricate /tʃ/. Hence, the good performance of our Creole-French bilinguals on the /s/-/ʃ/ French contrast could be explained by the presence of an affricate containing the /ʃ/ consonant in the phonemic inventory of Mauritian Creole.

It should be noted, however, that although the majority of studies examining bilinguals' performance on L2 phonemic contrasts reported difficulties, recent research focused on individual differences also reported native-like performance, not only in early bilinguals (Sebastián-Gallés and Baus, 2005; Sebastián-Gallés and Díaz, 2012) but also in late bilinguals (Díaz et al., 2012). For example, Sebastián-Gallés and Baus (2005) have tested early Spanish-Catalan bilinguals on the /e/-/ε/ contrast in various tasks, and observed that approximately 68% of participants fell within the accuracy range of Catalan native speakers in a categorization task. This is exactly what we observed in our study, in which only 33% of the bilinguals were less accurate than the native French speakers. Also, in a recent study conducted with late Dutch-English bilinguals, Díaz et al. (2012) reported that 43.6% of the bilinguals performed like native English speakers in a categorization task. In fact, both Sebastián-Gallés and Baus (2005) and Díaz et al. (2012) observed that it is in a task where lexical access is mandatory, like the lexical decision task in which participants have to discriminate between words and non-words, that most bilinguals (82 and 87% respectively) performed poorly compared to native speakers. More studies are thus required to examine whether difficulties with the French /s/-/ʃ/ contrast are more likely to occur in Creole-French bilinguals in other tasks like lexical decision.

In a second step of this study, we examined whether the way in which Mauritian Creole listeners categorize the /s/ and /ʃ/ consonants can be modified by introducing small changes in the information these speakers were given about the identity of the speaker. Globally, in comparison to Mauritian Creole listeners who have received no information about the identity of the speakers, Mauritian Creole listeners who were told that the stimuli were produced by a speaker expected to have both /s/ and /ʃ/ in his phonemic inventory because of his attributed professional activity, tended to give more *chaque* responses. Additional *post-hoc* analyses examining the differences in the percentage of *chaque* responses between the two groups of Creole-French listeners on each step of the continuum revealed that the bias toward the /ʃ/ consonant for the with-information group mainly occurred in the vicinity of the middle of the continuum, that is on step 7 containing almost equal proportions of the acoustics of /s/ and /ʃ/. Although we cannot have a direct access to the representation that participants created about the speaker, this representation is likely to be that of someone that produces the /s/-/ʃ/ consonant contrast, causing a bias toward the /ʃ/ consonant in the participants' responses. This contrast is clearly subject to linguistic insecurity. Corpus-based studies show that the /s/-/ʃ/ opposition is subject to variation in records of informal Mauritian Creole speech (Kriegel et al., in press). While monolingual speakers of Creole tend to systematically pronounce /s/ in contexts where the corresponding French item would have /ʃ/, speakers of urban Creole varieties in contact with French, like a bank employee with a French patronym, alternate between /s/ and /ʃ/ in the same phonetic environments. In another corpus-based study by Ludwig et al. (2009), the authors cite examples of hypercorrection in the pronunciation of lexemes that even in French contain the /s/ sound: [a waiter in a restaurant mostly acquainted with European tourists opens a bottle of wine saying [ʃãte] as opposed to the French form [sãte] “cheers” to his French speaking guests]. Our present findings thus suggest that a minor change in the instructions that participants received is sufficient to induce differences in the way speech sounds are categorized.

At a more theoretical level, our results are in accordance with the growing body of research showing that speech sound categorization is influenced by the representation that the listeners form about the speaker (e.g., Niedzielski, 1999; Strand, 1999; Hay et al., 2006; Hay and Drager, 2010; Drager, 2011). In a recent study, Hay et al. (2006) found that participants from New Zealand are more accurate in identifying vowels involved in a merging process, when these participants are shown a photograph of someone who was dressed as though he/she was from a higher social class, and thus as someone who is more likely to produce distinctly the two vowels. In our experiment, one possibility is that the socially valued job attributed to the speaker as well as the fact that he was said to work in the capital of Mauritius, have lead to the building up of a representation of someone that frequently speaks French and thus pronounces the /s/ and /ʃ/ consonants clearly and distinctly. As a result, for stimuli about halfway between the /s/ and /ʃ/ consonant, participants might have adopted the implicit strategy that the word presented was *chaque* because they were faced with the speech of someone that produces the /ʃ/ segment. These results thus constitute new evidence that information about the speaker's social characteristics can bias speech sound categorization.

To conclude, our results converge with recent studies (Sebastián-Gallés and Baus, 2005; Díaz et al., 2012) showing that it is possible to find native-like performance in bilinguals, at least when they have to categorize speech sounds on a continuum. They also add to the growing body of research (Niedzielski, 1999; Strand, 1999; Hay et al., 2006; Hay and Drager, 2010; Drager, 2011) arguing for the influence of information about the speaker's social identity in the categorization of speech sounds.

### Conflict of interest statement

The authors declare that the research was conducted in the absence of any commercial or financial relationships that could be construed as a potential conflict of interest.

## Footnotes

^1^Ideally, the same bilinguals would be tested in the two conditions. However, to avoid strategic processing due to the fact that participants likely would have noticed the change made in the instructions, we chose to test two groups of bilinguals from the same population and matched as closely as possible on age, age of exposure to French and level of education.

^2^The same pattern of results is found when we apply a log odds ratio transform to the proportion of *chaque* responses for each participant (see Díaz et al., 2012 for the same procedure).

^3^Note that there is no effect of group when we compare the performance of the with-information group with that of the monolingual group, as it is the case when we compare the performance of the no-information group with that of the monolingual group. Hence, our two bilingual groups showed native-like performance. However, because the with-information group for which information about the identity of the speaker was specified necessarily received instructions that were different from the monolingual group, the appropriate comparison to show native-like performance is between the no-information and the monolingual group since in this case the experimental design is exactly the same for the two groups (the two groups have received the same instructions with no information about the identity of the speaker). What is examined in this second step of our study is whether the simple fact of giving a very short piece of information about the identity of speaker suffices to modify the categorization of the /s/ and /ʃ/ consonants in Creole-French speakers for which native-like performance was already shown in the first step of this study.
